# The Menace of Mouth Infections

**Published:** 1917-11

**Authors:** Oliver T. Osborne

**Affiliations:** Professor of Therapeutics, Yale University School of Medicine, New Haven, Conn.


### THE MENACE OF MOUTH INFECTIONS

OLIVER T. OSBORNE, M.D.

Professor of Therapeutics, Yale University School of Medicine, New Haven, Conn.

There is no more impressive sequence than the presence in a patient of a disease or abnormal condition such as infected teeth or gums, and the later surgical eradication of the focus of infection; or, to make the sequence terse and logical, let us put it thus: patient; ailment; crowned, bridged or decayed teeth, inflamed gums, or pyorrhea, or all of these things; roentgenograms of these suspected areas demonstrating pus and degenerated bone; operative demonstration of pus sinuses and cheesy, brokendown bone; and lastly, but unfortunately not always, the cure of the patient.

In this age, in which the laity has acquired from periodicals a smattering of medical science, and therefore thinks it has a perfect right to criticize medical men and to ridicule what they term "medical fads," let me urge that the nearest relative of the patient be present at the dental operation, and be placed in close proximity to the foul tooth, foul crown, or foul pus that is extracted from the mouth of the loved one. I know of no more impressive demonstration to the ordinary layman, and he often becomes a hysterical enthusiast for the removal of infected teeth, or even of those that may possibly be infected.

### MENACE OF CROWNED TEETH

Cruelly awakening to the physician is the demonstration of a bacterium in the blood or in a distant lesion, and a late demonstration of the same germ in an extracted tooth or in the secretions of a pocket in the gum. I do not believe that there is a greater menace to health today than crowned and bridged teeth, to say nothing of imperfectly filled and dead teeth, and of pyorrhea alveolaris. Furthermore, infection of the tonsils and the sinuses adjacent to the nose must never be overlooked. If such infection has not caused symptoms, it will do so, and its eradication is the only safety.

From infected areas in the mouth many pathogenic organisms have been isolated, probably the most dangerous being the pneumococcus and the *Streptococcus viridans*. We do not know how many pneumonias, following or accompanying influenzal affections, occur, because pneumococci are being carried in the mouth. We do not know how many times irregularity, weakness and actual disease of the heart are due to germs harbored in the mouth. Neither do we know how frequently the *Streptococcus viridans* is the cause of heart disease or adds its fatal potency to an already established chronic disease.

We do not know much about the *Endamoeba buccalis*, but we do know that it is probably always accompanied by other germs, and especially by the pyogenic germs. Pyorrhea alveolaris is unquestionably due to infection, but this kind of infection and inflammation does not often occur unless there is degeneration of the gum tissues, and such degeneration most frequently occurs after forty years of age. It may be here noted, parenthetically, that chronic colitis, or at least decomposition in the colon, also occurs most frequently after the age of forty, and both of these conditions are very frequent in people in middle life. Is the intestinal disturbance a sequence of the mouth infection?

Connellan and King[1] have isolated a nonpus-forming diplococcus which they believe produces dangerous toxine. They find these germs in the crypts of tonsils, and patients suffering from arthritis, asthma, endocarditis, myocarditis or nephritis have been found to harbor these germs in their mouths. They advise producing an autogenous vaccine and using this for some time before such diseased tonsils are eradicated.

It is not my purpose either to offer or to discuss roentgenograms of damaged teeth and gums; but let me assert that no dentist should devitalize or attempt to fill the roots of a devitalized tooth, which is to be preserved, without the aid of roentgenograms; that every case of pyorrhea alveolaris, every suspicious root, and occasionally all crowned and bridged teeth should be subjected to the scrutiny offered by the roentgen ray. It is useless to agree on the removal of one infected tooth and possibly leave other infected teeth in the mouth of the patient, and yet such carelessness is repeatedly agreed to by both physician and dentist. Also it is difficult to compel a dentist to operate immediately on all infected areas. He does one thing at a time, and then takes up something else. What surgeon

---

[1] King, J. J.: Further Observations on the Connellan-King Diplococcus Throat Infections, The Journal A. M. A., Jan. 13, 1917, p. 91.

would remove one point of infection and leave half a dozen others, or even one other infected area?

There must be hearty medical cooperation between the physician and the dentist in order to decide, in any individual patient, what teeth shall be preserved, what teeth must be removed, and how best to treat infected gums; also what part a diseased condition of the patient plays in the mouth condition.

M. I. Schamberg,[2] a noted New York dental surgeon, says:

"Crown and bridge work, as well as all work upon pulpless teeth, should be conducted by men who would specialize in that work, and who would, through the medium of X-ray, satisfy themselves that they are not putting a beautiful superstructure on a weak foundation. Roentgenograms should be taken from time to time after the insertion of such pieces, to determine the state of health or disease of the hidden parts. Patients' mouths should be kept free from infection at all costs, even though it should mean the removal of every tooth in the head. If it can be done without sacrifice of the teeth, so much the better, but a toothless mouth is to be preferred to one containing a single focus that menaces the health of the patient."

It should also be urged that, however well a patient is, these germs of infection, wherever they may be, are ready to do him some injury whenever an acute infection attacks him, or when his own systemic strength is in any way reduced. They are all waiting an opportunity, and how can we excuse a physician for omitting to study his patient's mouth How can we excuse the dentist who waits until tomorrow, or next month, or six months hence before he removes a source of infection which he has recognized? Or how can we excuse a dentist for carelessly constructing bridges or crowns in the mouth of a young patient, knowing the danger from infection in the years

---

2 Schamberg, M. I.: Journal of the Allied Dental Societies, December, 1915.

to come? In other words, have we the right to crown to the gums, even if they are wonderfully well fitted? Have we the right to construct permanent bridges, when an aseptic bridge or a removable plate is more sanitary?

### IODIN INJURIOUS

I do not propose to discuss the treatment of pyorrhea alveolaris, but let me emphasize the fact that dentists use iodin too freely on the gums. A little pain here, or a little infection there, and the gum is injured by repeated applications of iodin. We know the value of iodin as a counter-irritant or as a germ destroyer. We also know that it destroys the vitality of tissues, and many a gum has been burned and blistered, and inexcusably made a region for more infection. We should remember that there are always germs in the mouth, many of them harmless, but also many ready to attack a broken mucous membrane or ready to be absorbed into the system through such a lesion. Therefore, care should be exercised not to injure the mucous membrane of the mouth or gums, especially when an infection is being treated.

I find that infected teeth and tonsils are frequent causes of thyroid disturbance, causing both hypersecretion and hyposecretion, and no medicinal treatment will effect a cure until the foci of infection have been removed. The absorbed toxins produced by the germs of infection and by the local suppuration often act peculiarly on the thyroid gland. The thyroid may be considerably enlarged, yet may give absolutely no symptoms of increased secretion and need not be cystic. Something irritates the gland, and then something else inhibits the result of its increased activity. This is shown by the surgical removal of the source of the mouth infection, and sometimes marked symptoms of hypersecretion immediately occur. I have seen three such instances. In one case the gland became greatly enlarged after the operation, exophthalmos occurred, and a tachycardia of from 140 to 160 developed. Before the operation absolutely no thyroid symptoms were present, except a slightly enlarged gland.

Not only the thyroid, but many other internal secreting glands of the body, are disturbed by the absorption of germs or toxins from the mouth. The suprarenals are, probably, especially acted on, as we have great changes in blood pressure in these patients. Many a patient with a very high blood pressure shows no arterial or kidney excuse, no intestinal toxemic excuse, and this high pressure persists, even when the diet is made rigidly vegetarian. Consequently it is self-evident that there is internal secretion disturbance. Whether the suprarenals hypersecrete, or the thyroid, and one or two other glands perhaps undersecrete, it is difficult to determine, but that these glands are disturbed there is no question. Also there are many instances of very low blood pressure in these patients, and again we may not know whether the toxins depress the nervous system, or the vasomotor center; or whether they stimulate the thyroid to increased secretion of vasodilator substance, or inhibit the suprarenals as to their vasopressor substance.

Not infrequently patients with glycosuria have mouth infection. These cases are likely to be without typical diabetic symptoms, namely, without polyuria, and without thirst. In these cases there may be pituitary disturbance, or suprarenal disturbance, or pancreatic disturbance, or the glycosuria may be caused by the absorption of something that interferes with the liver or with muscle metabolism.

We must recognize that germs may be present in and around gums, allowing dangerous absorption of their toxins, without any local manifestations of pus. This is true of some germs that cause arthritis.

Again, there can be no question that the constant absorption of irritants or poisons from the mouth can cause irritation of the kidneys and subsequent chronic nephritis, even if focal infection of the kidneys is not thus caused, and many a case of autogenous infection or purulent infection of the kidney occurs without any apparent tangible excuse. Probably these obscure cases occur mostly from concealed infections of the mouth, tonsils or one of the sinuses adjacent to the nose.

We do not know that this continued absorption of concealed irritants in the mouth for months and years may not cause arteriosclerosis. All sorts of gastrointestinal disturbances, such as hyperacidity and ulcer of the stomach, can be caused by such mouth infections. And let us remember that cancer of the stomach seems to be on the increase. Recently I saw in consultation a patient who made a profound impression on me. He was a man but little over forty, who had, before it was discovered, far advanced, inoperable cancer of the stomach, and besides several bridges, there was hardly a tooth in his mouth that was not crowned. He had been fussing with his teeth and visiting dentists for fifteen years or more.

Statistics prove that cardiovascular-renal disease is increasing enormously, and we know that, in the last twenty-five years crowns, bridges and pivoted teeth have also increased in a most extraordinary fashion. One has only to look into the mouth of every patient who enters one's office, of every patient who enters the hospital or the dispensary, to see how hard the dentists are working.

One of the saddest things that can happen to a person is infection of the blood with the *Streptococcus viridans*. This germ probably always gains an entrance through the mouth, and if we shoud cultivate the pus and secretions from the patients who have diseased mouths, we should probably be astonished to find the number of times the *Streptococcus viridans* was present. In the vast majority of cases the only hope for the patient with *Streptococcus viridans* is to eradicate it from the mouth before it infects his blood and causes malignant endocarditis.

The relation of mouth germs to arthritis deformans seems proved, hence eradication of the source of infection before the joints are affected should be the preventive treatment of today. To allow infection in the mouth to persist until this painful and crippling disease develops is inexcusable.

For nearly two years now, at a public tuberculosis institution of which I am chairman of the medical board, we have examined the mouths, corrected the teeth and removed sources of infection of every patient who entered the institution, and owing to this preventive treatment we have had less tonsilitis, fewer sore throats, less indigestion, and less influenza and colds than we had before we began these mouth investigations.

I think that both dentists and physicians have been very slow in discovering the harm done to patients by crowns and bridges, by leaving infected teeth in place and by improperly filled teeth. Physicians do not pay proper attention to, and many times do not even look into, the mouths of their patients. I look back with chagrin and shame as I think of the probable cause of the chronic invalidism of some of my patients of years ago. But with this admission of neglect in the past, I deplore the present necessity of being compelled to fight with both the patient and his or her dentist for the removal of infected foci in the mouth. The dentist frequently will not cooperate. He naturally dislikes to remove his own fine handiwork. Hence, I repeat, when a bad tooth comes out, or a dirty crown comes off, let the patient, or, if he is anesthetized, his nearest relative, smell of it. This is an educational propaganda.

CONCLUSIONS

1. Most unexpected tolerance to pyorrhea alveolaris and to tooth infection is found.

2. Chronic invalidism may be caused by mouth infections.

3. The blood pressure may be raised or lowered by mouth infections.

4. The thyroid gland is frequently enlarged and may hypersecrete or hyposecrete in these infections.

5. Serious disturbances of the blood, heart, kidneys, stomach, intestines and joints are frequent from mouth infections.

6. Glycosuria can be, and perhaps true diabetes mellitus may be, caused by mouth infections.

7. Serious distant focal infections may occur from mouth infection.

8. Serious brain and nerve disturbances, as well as neuritis, may occur from mouth infection.

9. Ulcer of the stomach, pyelitis, appendicitis and chronic colitis may be caused by pyorrhea alveolaris and mouth infection.

10. Pneumonia, especially that which follows influenza, may frequently be caused by pneumococci long carried in the patient's mouth.

11. No treatment of these conditions will be of any avail until the mouth is made clean.

12. Stock or autogenous vaccines are not very promising as to their therapeutic value, but in obstinate cases they should be tried. Therefore, it is generally well to grow a culture from the infection in the mouths of these patients, that autogenous vaccines may be made and used, if desired.

13. One should be very careful not to promise a cure of a distant condition, although that condition was caused by the mouth infection. However, many brilliant cures are caused by surgical eradication of infected areas. The patient should always be told that the surgical removal of the infected area in the mouth does not remove the germ localized in distant parts of the body, nor does it immediately cure an inflammation caused by these germs in a distant part, nor will it restore degenerated tissue, but it will remove the primary source of infection.

14. It should be urged that any fresh lesion of the mucous membranes of the mouth is a source of danger, much as is a lesion of the skin. The efficiency of the integument in warding off disease germs has long been recognized. It should be recognized that fresh cuts, abrasions and blistering of the mucous membrane of the mouth with iodin, or other strong escharotics, offer the opportunity for the absorption of germs that may be freshly received into the mouth, and more especially of germs already in the mouth.—*Journal A. M. A.*, October 20.

